# Machine Learning Models for Predicting In-Hospital Mortality in Acute Aortic Dissection Patients

**DOI:** 10.3389/fcvm.2021.727773

**Published:** 2021-09-17

**Authors:** Tuo Guo, Zhuo Fang, Guifang Yang, Yang Zhou, Ning Ding, Wen Peng, Xun Gong, Huaping He, Xiaogao Pan, Xiangping Chai

**Affiliations:** ^1^Department of Emergency Medicine, The Second Xiangya Hospital, Central South University, Changsha, China; ^2^Emergency Medicine and Difficult Diseases Institute, Central South University, Changsha, China; ^3^Trauma Center, Changsha, China; ^4^College of Information Science and Engineering, Hunan Normal University, Changsha, China

**Keywords:** acute aortic dissection, machine learning, extreme gradient boost, in-hospital mortality, prediction

## Abstract

**Background:** Acute aortic dissection is a potentially fatal cardiovascular disorder associated with high mortality. However, current predictive models show a limited ability to efficiently and flexibly detect this mortality risk, and have been unable to discover a relationship between the mortality rate and certain variables. Thus, this study takes an artificial intelligence approach, whereby clinical data-driven machine learning was utilized to predict the in-hospital mortality of acute aortic dissection.

**Methods:** Patients diagnosed with acute aortic dissection between January 2015 to December 2018 were voluntarily enrolled from the Second Xiangya Hospital of Central South University in the study. The diagnosis was defined by magnetic resonance angiography or computed tomography angiography, with an onset time of the symptoms being within 14 days. The analytical variables included demographic characteristics, physical examination, symptoms, clinical condition, laboratory results, and treatment strategies. The machine learning algorithms included logistic regression, decision tree, K nearest neighbor, Gaussian naive bayes, and extreme gradient boost (XGBoost). Evaluation of the predictive performance of the models was mainly achieved using the area under the receiver operating characteristic curve. SHapley Additive exPlanation was also implemented to interpret the final prediction model.

**Results:** A total of 1,344 acute aortic dissection patients were recruited, including 1,071 (79.7%) patients in the survivor group and 273 (20.3%) patients in non-survivor group. The extreme gradient boost model was found to be the most effective model with the greatest area under the receiver operating characteristic curve (0.927, 95% CI: 0.860–0.968). The three most significant aspects of the extreme gradient boost importance matrix plot were treatment, type of acute aortic dissection, and ischemia-modified albumin levels. In the SHapley Additive exPlanation summary plot, medical treatment, type A acute aortic dissection, and higher ischemia-modified albumin level were shown to increase the risk of hospital-based mortality.

## Introduction

Acute aortic dissection (AAD) is amongst the most common cardiovascular disorders; moreover, it is renowned for its high mortality (1, 2). Research has shown that ~1–2% of all patients with AAD die every hour following the onset of symptoms (3). Despite considerable improvements to the treatment of AAD recently, the in-hospital mortality of AAD remains at a concerningly high level (4)—nearly 20% of all patients with AAD die before hospital admission (5). Therefore, being able to predict the in-hospital mortality risk of this disorder precisely and efficiently in its early phase would undoubtedly improve the prognosis of patients diagnosed with AAD in the future.

Previous studies were unable to comprehensively detect the risk of in-hospital fatality or short-term death in patients with AAD (6–8). Tolenaar et al. (6) evaluated the death risk of this disorder and were able to produce a convenient bedside prediction tool for patients with acute type B aortic dissection. Similarly, Leontyev et al. (7) developed a scorecard to anticipate the short-term mortality of patients with type A AAD, which has proved to be very useful. In addition, Yang et al. (8) managed to identify potential predictors of in-hospital mortality, and subsequently constructed a predictive nomogram prototype to detect high-risk patients with AAD. However, these studies were analyzed using a conventional logistic regression (LR) method, which involves statistical assumptions about the independent linear relationship between the variables and the outcomes, or neglected the analysis of other valuable variables. Consequently, these findings are somewhat limited due to the complex process, inadequate predictive strength, and poor stability.

Machine learning (ML) is a specific form of artificial intelligence (AI) that automatically obtains valuable information and can recognize underlying patterns within large sets of data, subsequently generating an outcome prediction (9). Compared with traditional prediction methods, ML techniques perform on a superior level, and therefore, have been applied to an array of medical services, such as image identification, diagnosis, and treatment (10, 11). Existed research has evidenced the capability of ML algorithms to improve patient outcomes in relation to sepsis, based on the development of diagnosis and risk prediction models (12–14). Martinez et al. (15) also developed a ML model to identify high-risk patients for acute kidney injury at an early stage. Another recent study focused on patients presenting with chest pain in the emergency department, whereby it was found that ML had a critical role as a decision support tool for early detection of myocardial infarction (16). Therefore, the implementation of ML can be seen as a major contributor to improving patients' quality of life.

In recent times, ML has emerged in the context of aortic dissection. Huo et al. (17) successfully demonstrated the use of ML models to identify patients with AAD from misdiagnosed cases. This is a beneficial finding as it would aid early classification of the disorder and would enable timely decision-making by physicians. However, there is little research regarding the use of the ML algorithm to predict short-term outcomes of patients with AAD.

The purpose of this study was to construct and evaluate a ML model with the goal of predicting in-hospital mortality in patients with AAD. The significance of the results could aid efficient detection of high-risk patients and could effectively allocate appropriate medical resources upon AAD diagnosis.

## Materials and Methods

### Study Design and Setting

A retrospective single-center study was designed, whereby the clinical information of patients with AAD admitted to the Second Xiangya Hospital of Central South University were investigated. The patients were admitted to the hospital between January 2015 to December 2018. Prior to study commencement, ethical approval was granted by the institutional review board; as this was a retrospective observational study, the requirement for informed consent was removed.

Enrollment in the study involved 1,344 adult patients with AAD. Classification relied upon Stanford criteria, whilst magnetic resonance angiography (MRA) or computed tomography angiography (CTA) was used to diagnose AAD, based on the 2014 European Society of Cardiology (ESC) guidelines regarding the medical treatment and diagnosis of AAD (18). The exclusion criteria of the study included: (1) being under the age of 18; (2) the presence of intramural hematoma; (3) pregnancy; (4) hospital admission being ≥14 days since the commencement of symptoms.

### Features Extraction

Extraction from electronic medical registers involved the following features: age, sex, height, weight, body mass index, hypertension, diabetic status, stroke, atherosclerosis, Marfan syndrome, blood pressure, symptoms, smoking, drinking, Stanford classification, and treatment. These features were extracted based on the timeline of January 2015 to December 2018.

Relevant laboratory features were also collected, including: white blood cell count, neutrophil ratio (N%), lymphocyte ratio (L%), platelet count, hemoglobin, alanine transaminase, aspartate aminotransferase, albumin, total bilirubin, direct bilirubin, creatinine, blood urea nitrogen, uric acid, myoglobin, creatine kinase, creatine kinase-MB, troponin T, B-type natriuretic peptide, D-dimer, ischemia-modified albumin, C-reactive protein, erythrocyte sedimentation rate, procalcitonin, and lactate dehydrogenase. All variables were detected within the initial 24 h of patient admission; the main laboratory of the Second Xiangya Hospital was responsible for analysis of each variable.

### Clinical Outcome

In-hospital mortality was regarded as the clinical outcome, which referred to all causes of death during a period of hospitalization.

### Data Preprocessing

For the further development of ML models, categorical features were preprocessed according to their natures. For instance, the treatment of patients was encoded as 0, 1, or 2 (0 = Medical treatment, 1 = Endovascular treatment, 2 = Surgical treatment). Moreover, the type of AAD and gender were encoded as 1 or 2 (type of AAD: 1 = type A, 2 = type B; gender: 1 = male, 2 = female). In other cases, the features related to clinical conditions such as smoking, drinking, medical history, and symptoms are binary, were encoded as 0 or 1 (0 = absence, 1 = presence).

### Model Construction

Once the features were inputted, ML algorithms were applied, including LR, decision tree (DT), Gaussian naive bayes (GaussianNB), K nearest neighbor (KNN), and extreme gradient boost (XGBoost). These algorithms enabled predictions regarding in-hospital mortality in a sample of patients with AAD. Python programming software (version 3.6) was used to build the predictive models.

### Model Training and Performance Evaluation

**Figure 1:**
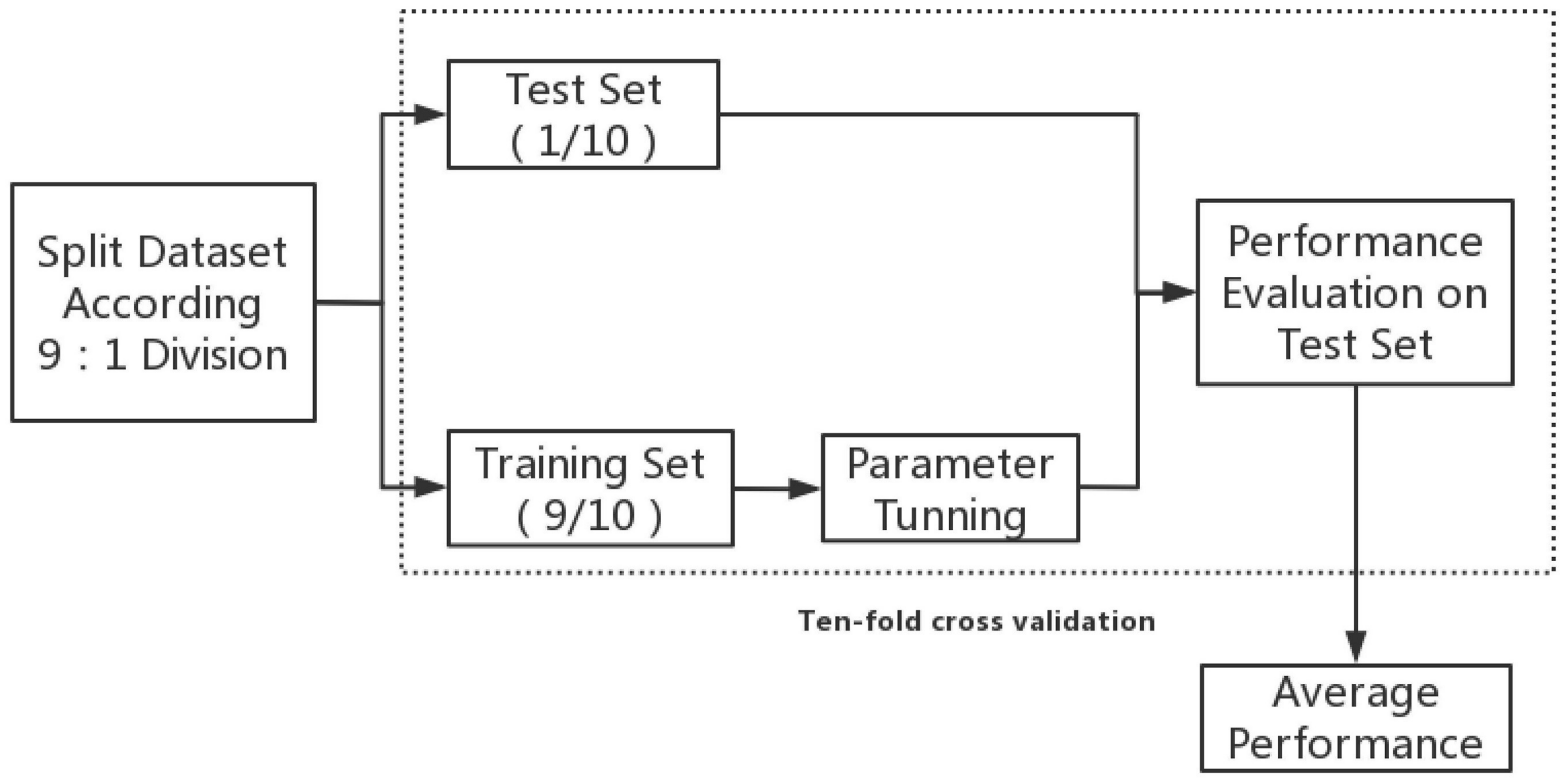
Training and evaluation procedures of machine learning model.

Figure 1 displays the concise training flow chart; firstly, all the data was randomly split into training and test sets based on 9:1 division. Optimal model parameters were then modified within the training set. To avoid overfitting, the model was tested on an independent test set that was unseen during training.

The model performance of the test set was evaluated by creating receiver operating characteristic (ROC) curves and, respectively, calculating the area under the ROC (AUROC) for all the models. At various thresholds, creation of the ROC utilized the “true positive rate” (TPR) against the “false positive rate.” Model capability was evaluated using the AUROC. To conduct a comprehensive assessment of model performance, the sensitivity (TPR), accuracy, average precision, specificity (true negative rate, TNR), positive predicted value (PPV), and negative predictive value (NPV) were all acknowledged.

Finally, 10-fold cross validation was implemented in the aforementioned procedures; this reduced the variability in estimations of model performance and ensured that the estimated performance of a model would reflect its practical performance. The model that obtained the best average performance metrics of the 10-fold-validation was then regarded as the optimal ML predictive model of in-hospital mortality in patients with AAD.

### Model Interpretation

There are distinctive Black-Box characteristics associated with ML, which can weaken the model's ability to produce correct interpretations (19). Nonetheless, the reasons behind each predictive outcome should be ascertained. To achieve this, in the ML model, the importance of each feature was evaluated by the feature importance score, which was determined based on the average reduction of loss when a feature was used as a partition attribute (20). The higher the feature score, the greater the influence of the feature on the prediction.

The SHapley Additive exPlanation (SHAP) method was used to expand and enhance the interpretation of the XGBoost model; the SHAP method provides a visualization of the prediction created by the final model. Concurrently, the DT model was also interpreted through SHAP Tree-Explainer as a way of comparing the results of SHAP. Cooperative game theory was initially used to establish the SHAP method; the theory was further developed to facilitate the SHAP method's ability to calculate the individual contribution values of each feature toward the final prediction (21). The SHAP method also evidenced the positive or negative influence of each feature value on the predicted results.

To explain the single prediction of the ML model, Local Interpretable Model-agnostic Explanations (LIME), a commonly used local explanation tool, was included in the model interpretation (22). LIME utilizes interpretability models, including linear models and tree-based models, to locally infer the target black box model's prediction. Although this does not result in significant depth within the model, this method is able to detect changes in the output of the black box model based on slight perturbations of the input. The model can then train an interpretability model at specific points of interest (the original input) based on this change. An important point to acknowledge is that the interpretability model is a local approximation of the black box model, as opposed to a global approximation, which also explains the origin of its name.

### Statistical Analysis

Two patient groups were constructed according to their status as deceased or alive during the hospitalization period. Variables were compared between these groups, whilst characteristics for continuous variables were shown as mean ± standard deviation or as median (IQR), and for categorical variables were shown as a percentage or frequency. Student's *t*-test (normal distribution) or Mann–Whitney U-test (skewed distribution) were used to compare continuous variables; in contrast, Fisher's exact test or Chi-square analysis were implemented to compare categorical variables. Regarding missing data, any feature with >10% of the data missing was eliminated; however, for features with <10% of the data missing, imputed values (which were combined using Rubin's rules) were used to impute the missing data. Statistical analyses were all completed using R software. Statistical significance was deemed when two-sided *P* < 0.05.

## Results

### Demographic Information and Clinical Characteristics

**Figure 2:**
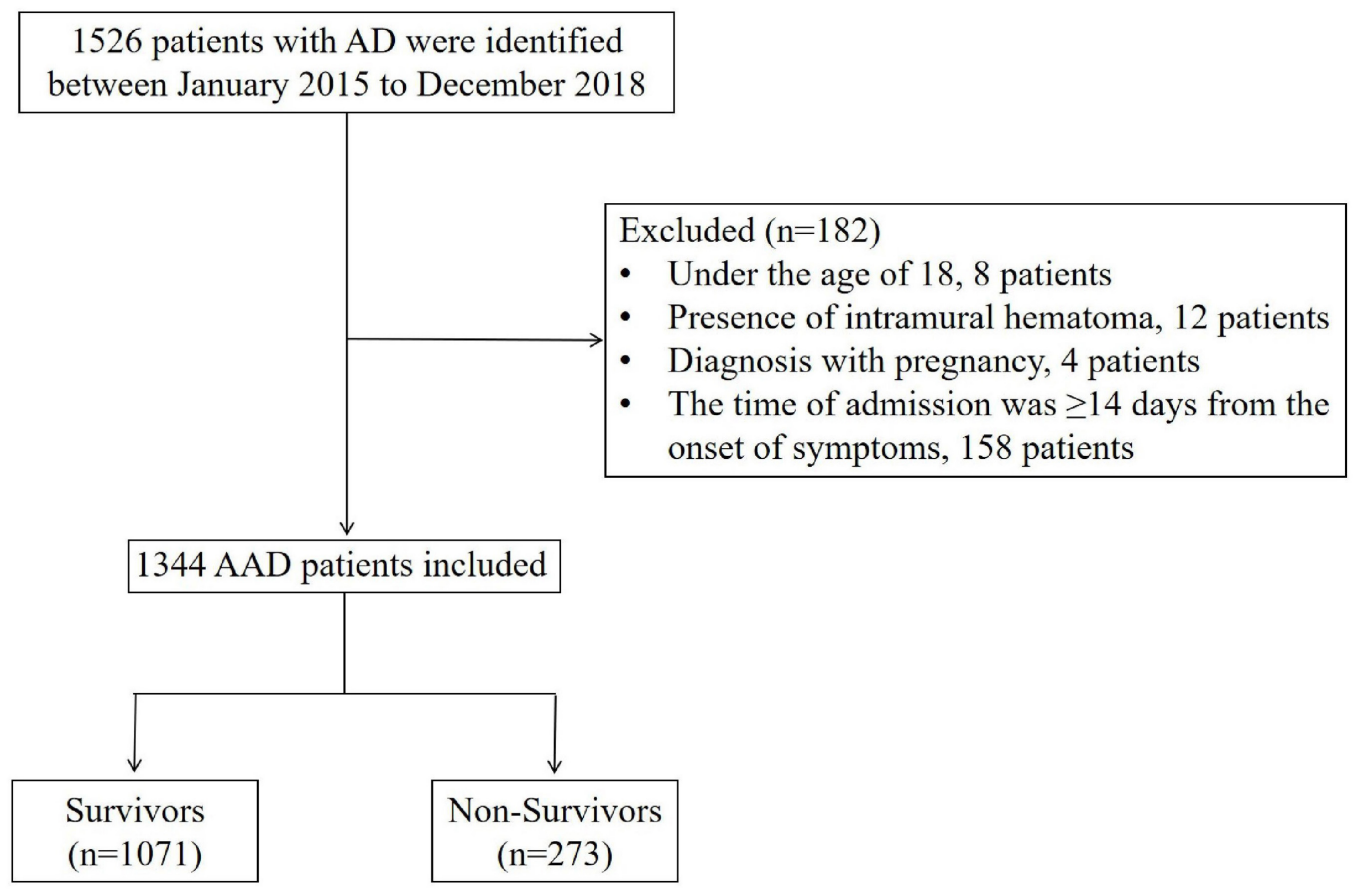
Flow chart of patient enrollment.

This study involved the recruitment of 1,344 patients with AAD between January 2015 to December 2018 (Figure 2). Table 1 shows the baseline characteristics of this patient sample, categorized based on survival status. Overall, 273 (20.3%) patients were classified as deceased, whilst 1,071 (79.7%) were classified as survived during hospitalization, with 662 (49.26%) type A acute aortic dissection (TA-AAD) patients and 682 (50.74%) type B acute aortic dissection (TB-AAD) patients, respectively. Among the cohort, 351 (26.12%) received medical treatment, 558 (41.52%) received endovascular treatment, and 435 (32.37%) underwent open surgery. The average age of the cohort was 52.37 ± 11.73 years; 80.36% of the patients were male, while 19.64% were female.

**Table 1:** Baseline characteristics of the AAD patients.

**Characteristics**	**Total** **(** ***n*** **= 1,344)**	**Survivors** **(** ***n*** **= 1,071)**	**Non-survivors** **(** ***n*** **= 273)**	***P*** **-value**
Age (years)	52.37 ± 11.73	52.36 ± 11.53	52.39 ± 12.50	0.975
Gender				0.915
Male	1,080 (80.36%)	860 (80.30%)	220 (80.59%)	
Female	264 (19.64%)	211 (19.70%)	53 (19.41%)	
**Physical examination**				
Height (cm)	167.70 ± 7.05	167.86 ± 6.85	167.04 ± 7.75	0.084
Weight (kg)	70.87 ± 14.11	70.97 ± 13.77	70.48 ± 15.39	0.609
BMI (kg/m^2^)	25.12 ± 4.33	25.11 ± 4.22	25.16 ± 4.75	0.870
SBP (mmHg)	147.22 ± 29.92	149.41 ± 28.29	138.65 ± 34.35	<0.001
DBP (mmHg)	82.17 ± 18.57	83.87 ± 17.78	75.51 ± 20.06	<0.001
Smoking	443 (32.96%)	364 (33.99%)	79 (28.94%)	0.113
Drinking	193 (16.47%)	159 (17.23%)	34 (13.65%)	0.314
**Medical history**				
Hypertension	949 (70.61%)	753 (70.31%)	196 (71.79%)	0.630
Diabetes	53 (3.94%)	43 (4.01%)	10 (3.66%)	0.790
Stroke	42 (3.12%)	30 (2.80%)	12 (4.40%)	0.176
Atherosclerosis	102 (7.59%)	79 (7.38%)	23 (8.42%)	0.559
Marfan syndrome	25 (2.35%)	16 (1.84%)	9 (4.64%)	0.049
**Laboratory results**				
White blood cells count (×10^9^/L)	11.70 ± 4.04	11.45 ± 3.87	12.66 ± 4.52	<0.001
Lymphocyte Ratio (%)	11.51 ± 6.18	11.82 ± 6.17	10.30 ± 6.09	<0.001
Neutrophil Ratio (%)	81.96 ± 8.13	81.66 ± 8.15	83.15 ± 7.97	0.007
Platelet count (×10^9^/L)	190.85 ± 82.32	195.20 ± 83.60	173.79 ± 74.81	<0.001
Hemoglobin (g/L)	125.25 ± 20.77	126.10 ± 19.59	121.90 ± 24.65	0.003
Alanine transaminase (μ/L)	21.50 (14.10–39.00)	20.90 (13.75–36.15)	24.00 (15.10–56.40)	<0.001
Aspartate aminotransferase (μ/L)	21.10 (15.90–35.10)	20.50 (15.55–32.45)	24.10 (17.80–55.50)	<0.001
Albumin (g/L)	35.62 ± 4.61	35.80 ± 4.65	34.92 ± 4.41	0.005
Total bilirubin (μmol/L)	17.36 ± 10.40	17.43 ± 10.68	17.11 ± 9.21	0.655
Direct bilirubin (μmol/L)	6.93 ± 6.62	6.90 ± 6.68	7.03 ± 6.35	0.775
Creatinine (μmol/L)	82.20 (66.00–113.93)	79.70 (65.20–106.15)	101.00 (71.50–155.70)	<0.001
Blood urea nitrogen (mmol/L)	6.51 (4.97–8.54)	6.22 (4.83–8.16)	7.78 (5.87–10.64)	<0.001
Uric acid (μmol/L)	319.55 (241.50–406.10)	319.00 ± 117.65	386.43 ± 158.89	<0.001
Myoglobin (g/L)	82.05 (50.08–202.90)	75.50 (48.70–165.00)	122.00 (65.20–394.00)	0.002
Creatine kinase (μ/L)	102.00 (59.80–196.15)	95.00 (56.45–182.60)	128.30 (72.00–352.10)	<0.001
Creatine kinase-MB (μ/L)	12.40 (8.07–17.90)	12.30 (8.30–17.50)	13.40 (7.30–21.90)	<0.001
Troponin T (pg/mL)	8.93 (3.89–20.79)	8.25 (4.06–16.60)	15.96 (3.52–47.36)	<0.001
B-type natriuretic peptide (pg/mL)	239.60 (104.00–673.45)	221.00 (94.55–542.50)	390.60 (155.00–1130.00)	<0.001
D-Dimer (mg/L)	3.79 (2.22–9.01)	3.56 (2.06–7.52)	5.94 (3.07–18.90)	<0.001
Ischemia-modified albumin (μ/ml)	75.25 ± 21.68	69.40 ± 15.31	84.41 ± 23.10	<0.001
C-reactive protein (μg/L)	52.80 (11.20–114.25)	61.40 (13.60–121.50)	19.50 (7.43–86.80)	<0.001
Erythrocyte Sedimentation Rate (mm/h)	22.00 (7.00–52.00)	23.00 (8.00–53.00)	17.00 (7.00–44.00)	0.070
Procalcitonin (ug/L)	0.17 (0.06–0.65)	0.16 (0.06–0.60)	0.25 (0.09–0.95)	0.382
Lactate Dehydrogenase (U/L)	243.00 (201.40–311.70)	240.60 (198.60–302.20)	263.20 (209.70–367.00)	<0.001
Symptom				0.058
Chest pain	1,089 (81.03%)	864 (80.67%)	225 (82.42%)	
Back pain	60 (4.46%)	47 (4.39%)	13 (4.76%)	
Abdominal pain	93 (6.92%)	81 (7.56%)	12 (4.40%)	
Syncope	19 (1.41%)	11 (1.03%)	8 (2.93%)	
Other	83 (6.18%)	68 (6.35%)	15 (5.49%)	
Type of AAD (Stanford)				<0.001
A	662 (49.26%)	436 (40.71%)	226 (82.78%)	
B	682 (50.74%)	635 (59.29%)	47 (17.22%)	
Treatment				<0.001
Medical treatment	351 (26.12%)	132 (12.32%)	219 (80.22%)	
Endovascular treatment	558 (41.52%)	546 (50.98%)	12 (4.40%)	
Surgical treatment	435 (32.37%)	393 (36.69%)	42 (15.38%)	

*BMI, body mass index; SBP, systolic blood pressure; DBP, diastole blood pressure*.

### Comparison of Baseline Characteristics Between Survivor and Non-survivor Groups

An insignificant difference was determined between age, gender, height, weight, and body mass index between the survivor and non-survivor groups (*P* > 0.05). However, the non-survivor group presented lower systolic (*P* < 0.001) and diastolic (*P* < 0.001) blood pressure at admission, compared with the survivor group. Furthermore, the non-survivor group showed statistically significantly higher levels of creatinine (*P* < 0.001), uric acid (*P* < 0.001), myoglobin (*P* = 0.002), creatine kinase (*P* < 0.001), troponin T (*P* < 0.001), B-type natriuretic peptide (*P* < 0.001), D-dimer (*P* < 0.001), and ischemia-modified albumin (*P* < 0.001), compared with the survivor group. Meanwhile, the platelet count (*P* < 0.001) and c-reactive protein (*P* < 0.001) values were lower in the non-survivor group compared with the survivor group. In addition, greater frequencies of Stanford type A AAD (*P* < 0.001) and Marfan syndrome (*P* = 0.049) were detected in the non-survivor group compared with the survivor group. It was also determined that the non-survivor group was more likely to receive medical treatment (*P* < 0.001) (Table 1).

### Predictive Performance of Five Models

**Figure 3:**
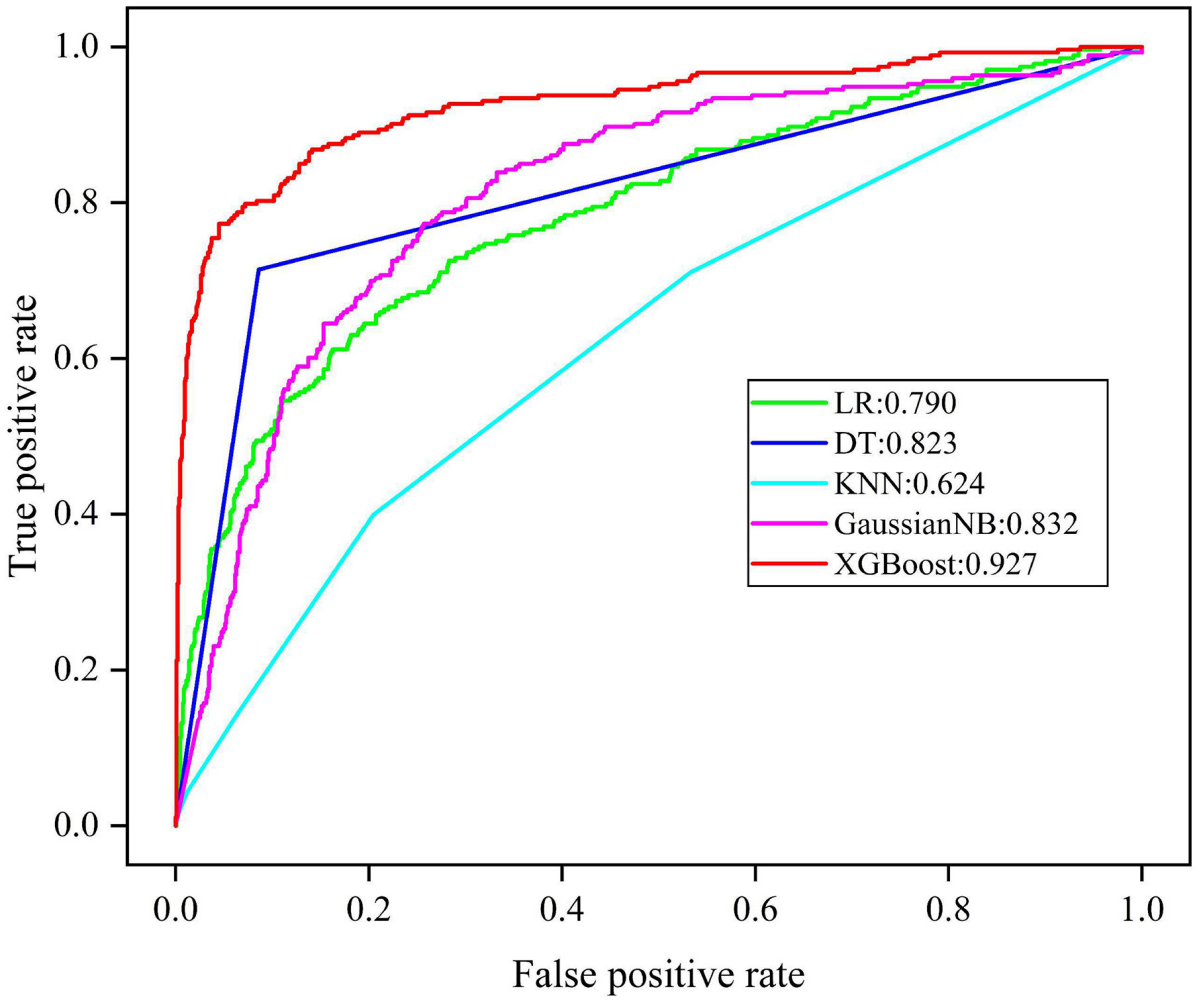
ROC analysis results of five models. LR, logistic regress; DT, decision tree; KNN, K nearest neighbor; GaussianNB, Gaussian naive bayes; XGBoost, extreme gradient boost.

Regarding the five derived models, the ROC curves of each are displayed in Figure 3, where the XGBoost model shows the largest mean AUROC of 0.927 (95% CI: 0.860–0.968) across 10 iterations. This is followed by the GaussianNB model of 0.832 (95% CI: 0.769–0.895), the model of DT 0.823 (95% CI: 0.779–0.867), the LR model of 0.790 (95% CI: 0.705–0.875), and the KNN model of 0.624 (95% CI: 0.545–0.703).

Since AUROC alone is an insufficient form of evaluation, the accuracy, average precision, TPR, TNR, PPV, and NPV were explored to comprehensively evaluate the performance of each model. The mean performance evaluation metrics are shown in Table 2, for each of the five ML models. Following comparison, XGBoost produced the most superior predictive performance, with an accuracy of 0.918 (95% CI: 0.838–0.998), average precision 0.683 (95% CI: 0.400–0.966), TPR of 0.729 (95% CI: 0.457–1.000), TNR of 0.966 (95% CI: 0.908–1.000), PPV of 0.855 (95% CI: 0.627–1.000), and NPV of 0.934 (95% CI: 0.869–0.999).

**Table 2:** Performance comparison between the five models.

**Algorithms**	**AUROC** **(95% CI)**	**Accuracy** **(95% CI)**	**Average Precision** **(95% CI)**	**Sensitivity** **(95% CI)**	**Specificity** **(95% CI)**	**PPV** **(95% CI)**	**NPV** **(95% CI)**
LR	0.790 (0.705–0.875)	0.837 (0.762–0.912)	0.387 (0.192–0.582)	0.334 (0.770–0.590)	0.965 (0.906–1.000)	0.743 (0.386–1.000)	0.851 (0.798–0.904)
DT	0.823 (0.779–0.867)	0.874 (0.825–0.923)	0.549 (0.394–0.704)	0.718 (0.490–0.946)	0.914 (0.874–0.954)	0.682 (0.569–0.795)	0.928 (0.874–0.983)
KNN	0.624 (0.545–0.703)	0.775 (0.725–0.825)	0.233 (0.173–0.293)	0.147 (0.380–0.283)	0.935 (0.887–0.983)	0.371 (0.115–0.627)	0.811 (0.781–0.841)
GaussianNB	0.832 (0.769–0.895)	0.813 (0.770–0.856)	0.300 (0.204–0.396)	0.231 (0.108–0.354)	0.961 (0.933–0.989)	0.599 (0.350–0.848)	0.831 (0.804–0.858)
XGBoost	0.927 (0.086–0.968)	0.918 (0.838–0.998)	0.683 (0.400–0.966)	0.729 (0.457–1.000)	0.966 (0.908–1.000)	0.855 (0.627–1.000)	0.934 (0.869–0.999)

*LR, logistic regress; DT, decision tree; KNN, K nearest neighbor; GaussianNB, Gaussian naive bayes; XGBoost, extreme gradient boost; TPR, true positive rate; TNR, true negative rate; PPV: positive predicted value; NPV: negative predictive value*.

### Model Interpretation

**Figure 4:**
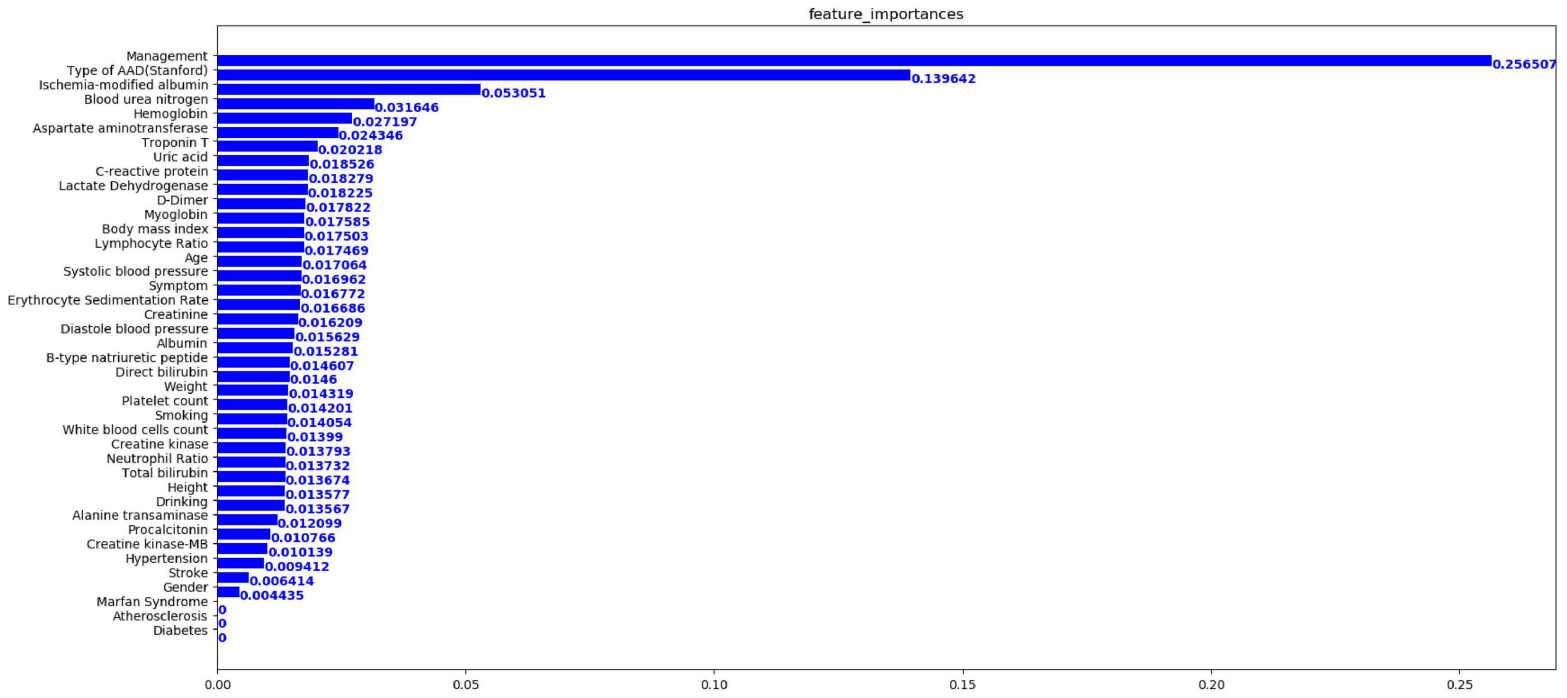
Importance matrix plot of the XGBoost model. The top 20 important features regarding the development of the final predictive model are depicted.

In terms of the XGBoost model, Figure 4 presents the feature importance matrix plot, whereby the importance of the features is determined in terms of creating the final predictive model. Figure 4 shows that the three most important features were treatment, type of AAD, and ischemia-modified albumin levels.

**Figure 5:**
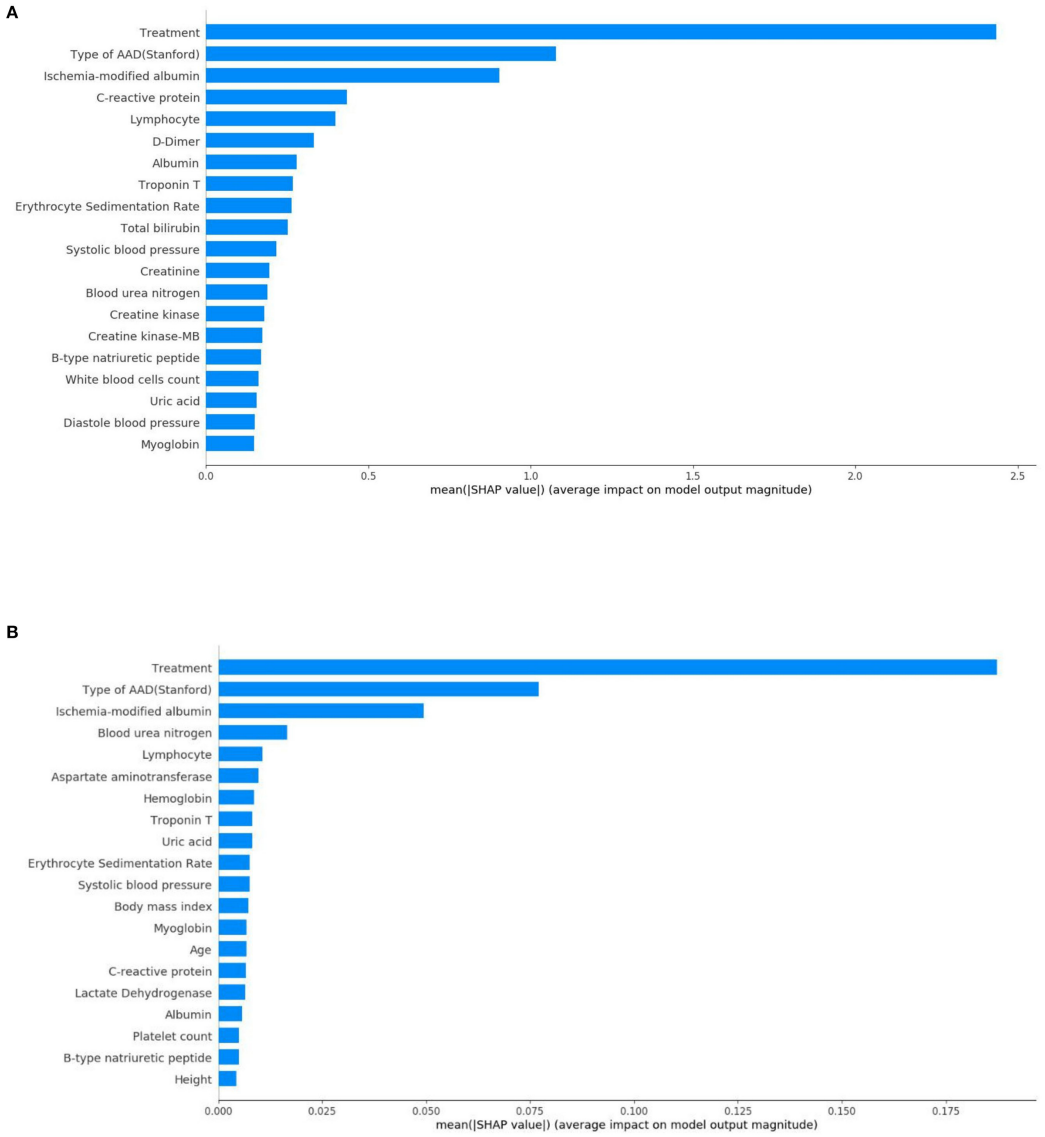
Standard bar charts of the average absolute SHAP values of each feature in the XGBoost model **(A)** and DT model **(B)**.

**Figure 6:**
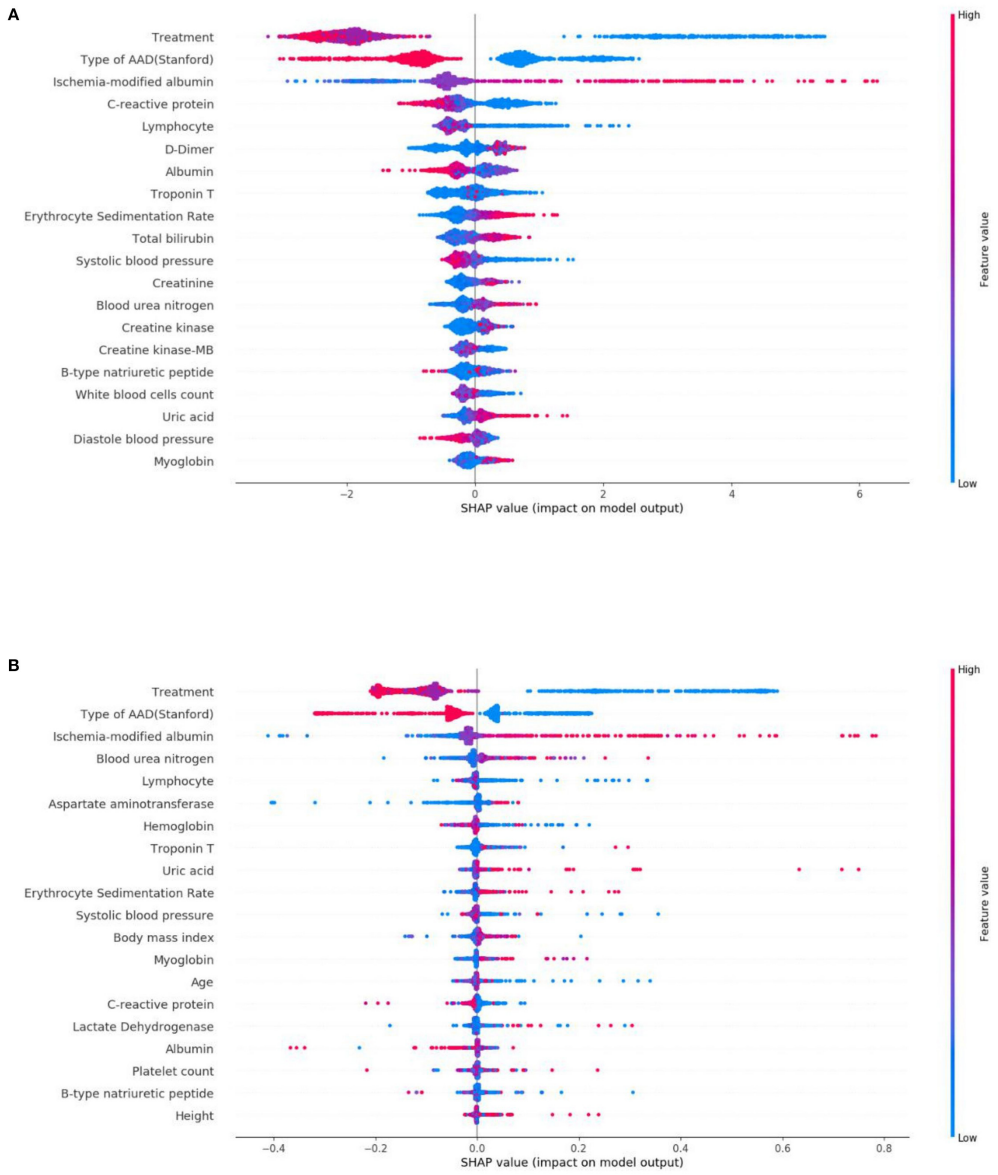
SHAP summary plots of the top 20 features in the XGBoost model **(A)** and DT model **(B)**. The higher the SHAP value of a feature, the higher the risk of in-hospital mortality. A dot is shown for each feature attribution value for the model of each patient, and thus, one patient is allocated a single dot for each feature. Dots are colored based on the values of the features for the respective patient and accumulate vertically to depict density. Treatment was divided into three categorical features: medical treatment (blue), endovascular treatment (purple), and surgical treatment (red). Type of AAD (Stanford) was divided into two categorical features: Type A (blue) and Type B (red). For continuous features, red represents higher feature values, whilst blue represents lower feature values.

From this, Figure 5 shows the average absolute SHAP values pertaining to the 20 most important features in the XGBoost model (Figure 5A) and the DT model (Figure 5B). As with the ranking of the feature importance scores, in both models, the top three features of the mean absolute SHAP values remained as treatment, type of AAD, and ischemia-modified albumin levels. The SHAP summary plots of the XGBoost model (Figure 6A) and the DT model (Figure 6B) explain the relationship between the feature type or level and the SHAP values. By comparing the SHAP results of XGBoost and DT model, an association was determined between positive SHAP values and medical treatment, type A AAD, and higher ischemia-modified albumin levels; resultantly, this denotes an increased risk of in-hospital mortality. Contrastingly, negative SHAP values were associated with endovascular or surgical treatment, type B AAD, and lower ischemia-modified albumin levels, which implies decreased risk of in-hospital mortality.

**Figure 7:**
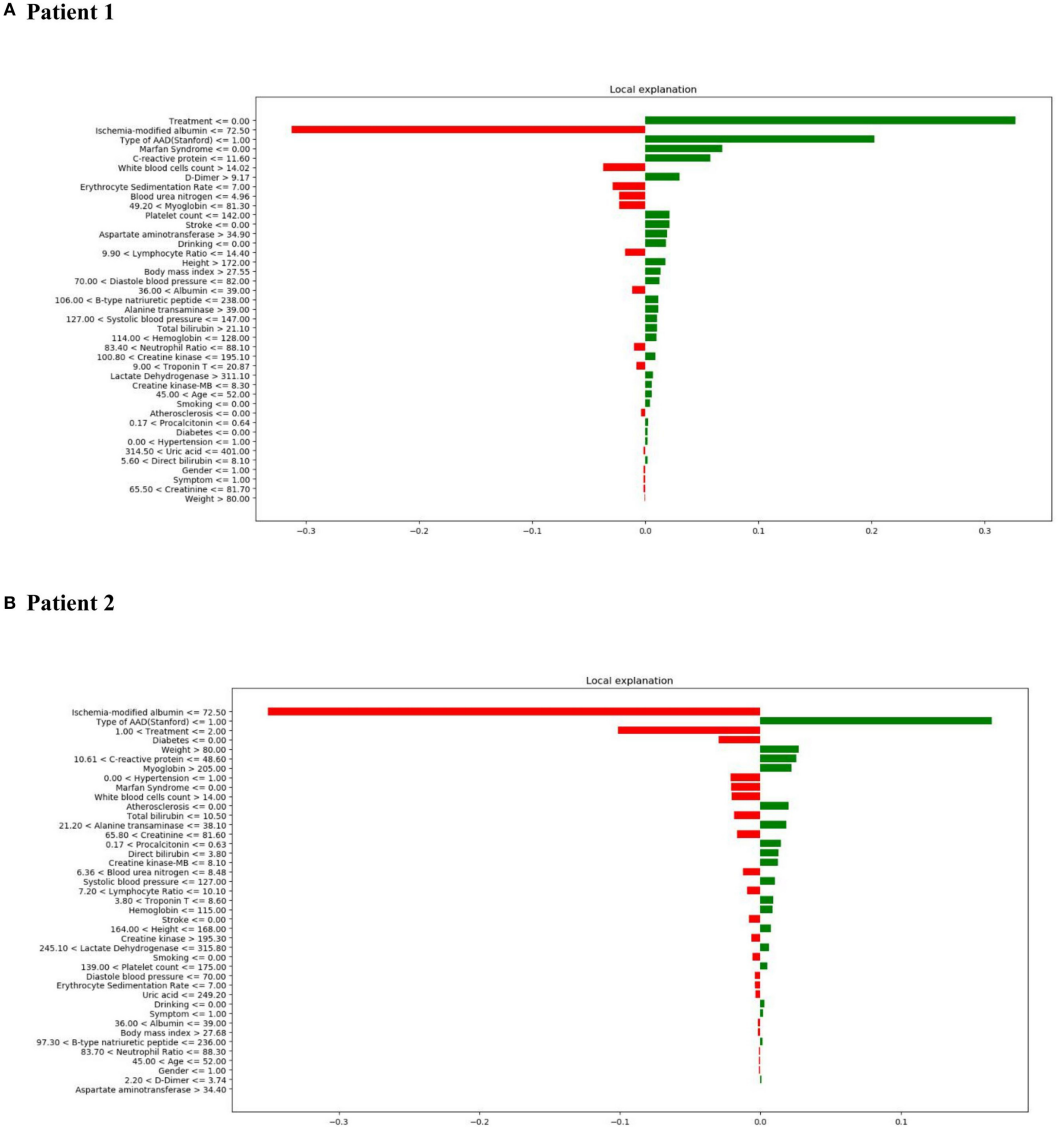
LIME results with XGBoost classifiers used for two patients with correct predictions (one true positive [in-hospital mortality] patient and one true negative [survival] patient). The contribution of various features to the likelihood of in-hospital mortality are shown for each patient. Each column illustrates the contributions of the features to the probability included in the models. Green represents a positive contribution and red represents a negative contribution. Below the feature, the depicted value signifies the weight coefficient provided by LIME, which is linked to the feature's contribution to the model prediction. **(A)** LIME explanation for true positive patient 1; **(B)** LIME explanation for true negative patient 2.

LIME was employed to explore the feature contributions of the predictions. The test dataset comprised two patients, whereby correct predictions had been formulated by the XGBoost model. Figure 7A, shows the correct prediction of in-hospital mortality pertaining to patient 1 from the “True Positive” group; this prediction was formed based on the patient receiving medical treatment, and having TA-AAD without Marfan syndrome association. In the “True Negative” group, patient 2 was also correctly predicted as survival (Figure 7B). The data for patient 2 showed low ischemia-modified albumin level (<=72.50 μ/ml), surgical treatment, and no indication of diabetes, all of which aided the negative prediction.

**Figure 8:**
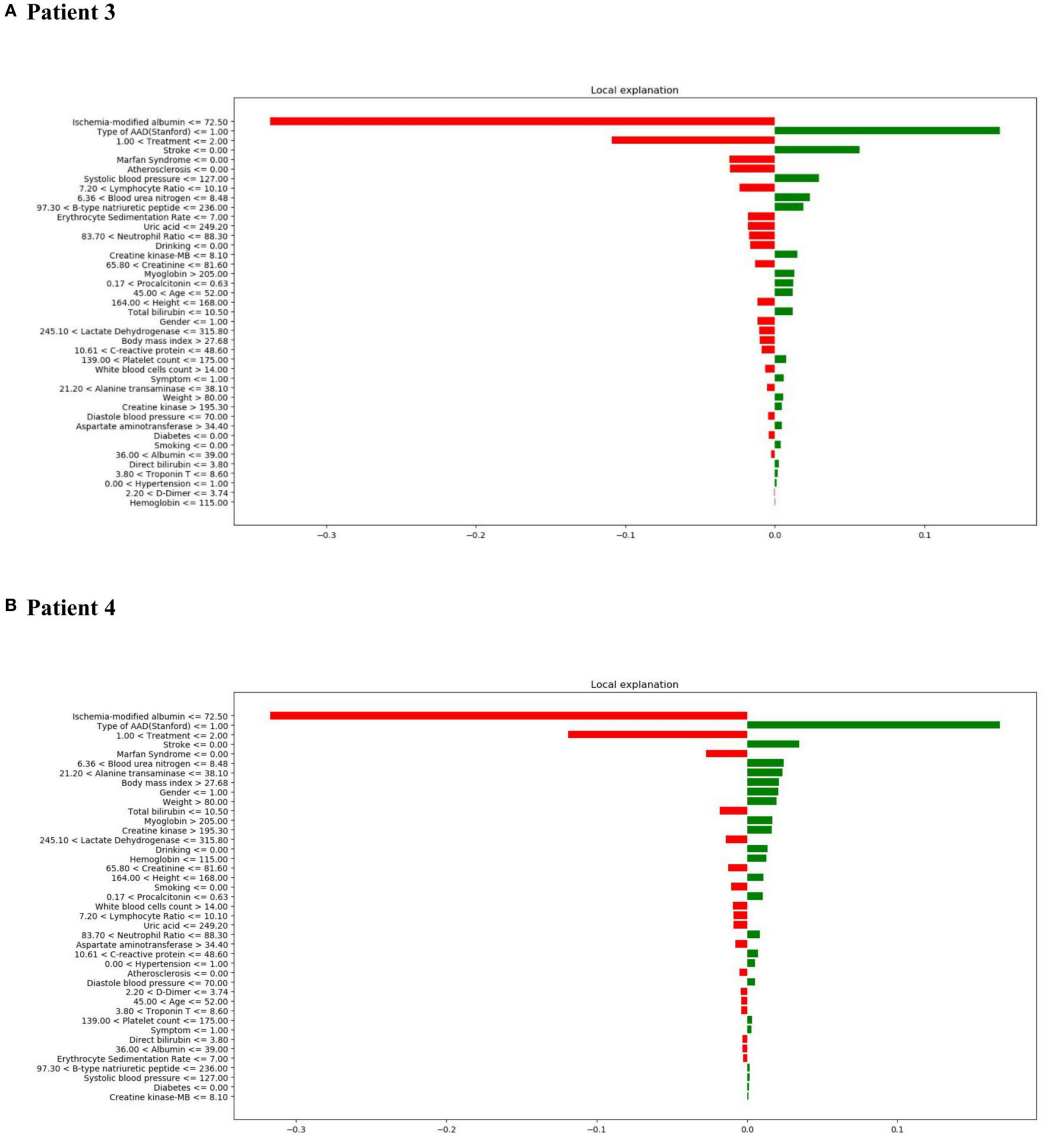
LIME results with XGBoost classifiers used for two patients with incorrect predictions (one in-hospital mortality patient [incorrectly predicted to have high probability of survival] and one survival patient [incorrectly predicted to have a high probability of in-hospital mortality]). **(A)** LIME explanation for false positive patient 3; **(B)** LIME explanation for false negative patient 4.

It is important to understand the reason behind incorrect interpretations. Therefore, patient 3 was included with a “False Positive” prediction—this patient was an in-hospital mortality patient who was incorrectly predicted to have a high likelihood of survival. Diagnosis of TA-AAD, the occurrence of stroke, and low systolic blood pressure (<=127.0 mmHg) were deemed to be the most influential features leading to the XGBoost model's prediction error (Figure 8A). In addition, patient 4 was included with a “False Negative” prediction, who was a survival patient but incorrectly predicted to have a high probability of in-hospital mortality (Figure 8B). It was found that low ischemia-modified albumin level (<=72.50 μ/ml), surgical treatment, and the presence of Marfan syndrome chiefly led to the prediction error in the XGBoost model.

In general, the interpretation results of SHAP and LIME are consistent. They show that the three most important characteristics that impact on the in-hospital mortality risk of patients with AAD are treatment strategy, type of AAD, and ischemia-modified albumin levels.

## Discussion

To date, this study is the first of its kind to apply ML to predict in-hospital mortality of patients with AAD. The study comprised 41 relevant features, whereby five ML models were successfully trained and developed to predict the in-hospital mortality risk of a cohort of patients. Out of the five models, the XGBoost model exhibited the best performance and the greatest AUROC for single-model prediction. Moreover, the predictions generated by the XGBoost model were deemed to be more reliable and accurate than conventional LR. Furthermore, the treatment strategy, type of AAD, and ischemia-modified albumin levels were identified as the most important variables linked to the prognosis of patients with AAD.

Recently, evidence has identified several factors that could be used to determine the risk of poor outcomes of AAD; however, their use as predictive factors of in-hospital mortality remains controversial (6–8). In addition, traditional LR presents a relatively weak indicator of predictive performance, such as the use of AUROCs, or can produce a higher probability of error compared with ML (23, 24).

As an adjusted dispersed gradient boosting library, XGBoost transforms the set of weak learners (25) to strong learners by implementing ML under the Gradient Boosting framework (26). In this present study, XGBoost produced outstanding prediction performance in the context of in-hospital mortality of a patient sample with AAD. A previous study found that the superior prediction performance of XGBoost can facilitate risk discrimination and early treatment of patients with acute kidney injury with mortality risk (27). Additionally, the XGBoost model has been shown to be capable of processing large datasets and can further analyze complex relationships between variables; in patients with sepsis, this model showed better prognosis prediction ability compared with traditional LR model (28). Although several ML models were tested to predict the mortality risk of patients with AAD in this study, the XGBoost model showed the most promising performance, which corroborates the findings of previous studies. Thus, clinicians and other relevant medical staff could make effective and individualized therapeutic strategies based on the predictive results of the XGBoost model, which would also facilitate more reasonable distribution of medical resources and would minimize the oftenexcessive medical costs faced by patients.

To the best of current knowledge, the primary treatment strategy for AAD is dependent on the type of AAD diagnosed for each patient. Approximately 1–2% of TA-AAD patients who do not receive any form of therapy will die every hour during the first 24 h of hospitalization, whilst almost 50% of patients will die within a week (4). Furthermore, the mortality rate can reach 20% within the first day due to severe complications, such as proximal or distal extension, valvular dysfunction, rupture, and pericardial tamponade; this can increase to 30% in the first 2 days (29). Over time, data from the International Registry of Acute Aortic Dissections (IRAD) has illustrated that the in-hospital mortality rate of patients with TA-AAD who undergo ascending-aortic-repair surgery has decreased significantly from 25 to 18%. However, patients who received medical treatment without surgery continued to face a high in-hospital mortality rate of 57% (30). For a long period, medical therapy alone was recommended as the main treatment strategy for uncomplicated TB-AAD, whilst thoracic endovascular aortic repair (TEVAR) was recommended for complicated TB-AAD (18). Yet, as clinical theory has continued to advance and medical techniques have become more innovative, the most appropriate therapy strategy for TB-AAD remains controversial, with endovascular techniques being used more for initially uncomplicated cases of TB-AAD. A randomized investigation found an association between endovascular stent-grafting technique and positive aortic remodeling, whereby a reduction of 5-years in the mortality risk was established compared with traditional medical treatment (31). Also, a considerable number of long-term follow-up studies have corroborated the finding that endovascular treatment is a better option for uncomplicated TB-AAD, based on the ability of early TEVAR to prevent the occurrence of fatal cardiovascular complications (31–33). Therefore, it is reasonable to suggest that patients with TA-AAD and patients with TB-AAD should receive immediate intervention upon diagnosis, including surgical repair or TEVAR, as this has been proven to improve patient prognosis drastically.

Under normal circumstances, the Stanford classification is used to determine the type of AAD: Type A indicates dissections involving the ascending aorta, as opposed to Type B which indicates dissections of the descending aorta (18). Based on previous findings, the anatomical categorization of AAD is the main indicator of a patient's prognosis (34). Under most conditions, TA-AAD is associated with wider involvement of dissection and an increasingly complicated pathologic change than TB-AAD. In a study conducted by Roselli et al. (35), it was found that over 40% of patients with AAD involving the proximal aorta died immediately, with an hourly mortality rate of 1–3% of patients. Findings from an IRAD investigation of patients with TA-AAD showed that the overall in-hospital mortality was 22%, while in patients with TB-AAD, the overall in-hospital mortality was 12–14% (30). In the present study, Stanford Type A dissections also showed a strong association with a higher risk of in-hospital mortality compared with Stanford Type B dissections. Hence, patients diagnosed with TA-AAD were in more critical conditions and had a greater likelihood of dying during hospitalization, possibly due to the involvement of the ascending aorta.

Ischemia-modified albumin was derived from altered the N-terminus of albumin following exposure to ischemic tissues. This led to a decrease of metal binding ability (36). Currently, although ischemia-modified albumin has been mainly used for diagnostic and prognostic purposes relating to acute coronary syndrome, elevated levels have also been linked to a poor outcome of patients experiencing acute chest pain and severe sepsis (37, 38). Moreover, a recent study depicted that raised ischemia-modified albumin levels corresponded with a high risk of in-hospital death in patients with AAD; this finding was consistent with the results of the current study (39). Elevated ischemia-modified albumin levels indicate ischemia-reperfusion and oxidative stress (40), the mechanism of which has been suggested by several studies. Firstly, the involvement of related arteries in the dissection of patients with AAD means that organ ischemia can raise ischemia-modified albumin levels. Secondly, unstable hemodynamical conditions caused by systemic tissue hypoxia could also be reflected in elevated ischemia-modified albumin. In general, these proposals imply that ischemia-modified albumin is a biomarker of an upstream process linked to the prognosis of patients with AAD, and therefore, elevated serum ischemia-modified albumin levels should be considered carefully and seriously.

Despite the many promising results of this study, the study design was somewhat inadequate. Not only was a small dataset used in this study, but it was also collected from a single source, thereby risking bias in the results. However, the source of the data, Second Xiangya Hospital, is an extensive medical center that provides care to the highest number of hospitalized patients with AAD in the Hunan Province. Therefore, the data retrieved from this site and used in this study is considered to be representative and reliable. A further restriction was that the features were manually recovered from electronic medical registers, which conveys a high likelihood of introducing observational error. Consequently, future studies should consider creating a real-time electronic record system that can record information rapidly and accurately. This study is also limited as the results cannot be applied to other nationalities or ethnicities, as the patients were all Chinese. Therefore, the generalized effectiveness of the model will need to undergo external testing before it can be deemed universally applicable. Nonetheless, the findings of this study will be useful to improve existing predictive models in future research; in particular, multi-center data should be included and external tests should be conducted rigorously regarding the predictions. Finally, to fully determine the black-box nature of the ML model, this study followed several previous studies (22, 41, 42) by using the SHAP method for global interpretation and LIME for local interpretation. Although the results of both types of interpretation of the XGBoost model were consistent and credible, improved robustness could be attained by using other interpretation methods, such as Shapley Lorenz, which is a novel global interpretation method that provides a global normalized measure of explainability. This would help to better explain our prediction model (43) and would improve the quality of future research.

## Conclusions

Overall, an XGBoost model was successfully developed to predict in-hospital mortality in patients with AAD; this is a novel achievement. This model is clinically significant as it provides a reliable early-risk assessment tool for clinicians and other relevant health care professionals. The main outcome of the results is that selection of treatment strategies, the type of AAD, and ischemia-modified albumin levels are the most crucial factors to determine in-hospital mortality predictions of AAD.

## Data Availability Statement

The data statement should be revised as: The data presented in the study are deposited in the Figureshare repository, accession number 16437960 (https://doi.org/10.6084/m9.figshare.16437960.v1).

## Ethics Statement

The studies involving human participants were reviewed and approved by the Ethics Committee of the Second Xiangya Hospital, Central South University (Changsha, China) (NO. LYF2020044). Written informed consent was not required for this study, in accordance with the local legislation and institutional requirements.

## Author Contributions

TG and ZF: study design. XC and TG: project administrative and support. TG, GY, YZ, HH, and XP: clinical data collection. TG and ZF: data analysis and machine learning development. TG: writing—original draft. TG, ND, GY, WP, XG, and YZ: writing—review and editing. All authors contributed to the article and approved the submitted version.

## Funding

This study was supported by Key Research and Development Program of Hunan Province, China (2019SK2022 to XC).

## Conflict of Interest

The authors declare that the research was conducted in the absence of any commercial or financial relationships that could be construed as a potential conflict of interest.

## Publisher's Note

All claims expressed in this article are solely those of the authors and do not necessarily represent those of their affiliated organizations, or those of the publisher, the editors and the reviewers. Any product that may be evaluated in this article, or claim that may be made by its manufacturer, is not guaranteed or endorsed by the publisher.
